# Study on the Function and Mechanism of Lin28B in the Formation of Chicken Primordial Germ Cells

**DOI:** 10.3390/ani11010043

**Published:** 2020-12-28

**Authors:** Qisheng Zuo, Jing Zhou, Man Wang, Yani Zhang, Guohong Chen, Bichun Li

**Affiliations:** 1 Key Laboratory of Animal Breeding Reproduction and Molecular Design for Jiangsu Province, College of Animal Science and Technology, Yangzhou University, Yangzhou 225009, China; 006664@yzu.edu.cn (Q.Z.); zhoujing980111@163.com (J.Z.); Zqs081901427@126.com (M.W.); ynzhang@yzu.edu.cn (Y.Z.); ghchen@yzu.edu.cn (G.C.); 2 Joint International Research Laboratory of Agriculture and Agri-Product Safety of the Ministry of Education of China, Yangzhou University, Yangzhou 225009, China

**Keywords:** primordial germ cells, *Lin28B*, gene function, *let-7a*, miRNA

## Abstract

**Simple Summary:**

In this study, we explored the function and molecular mechanism of *Lin28B* in the formation of chicken primordial germ cells (PGCs) in detail. Our results indicate that *Lin28B* participates in the formation of PGCs through *let-7a-3p*, which set a theoretical foundation for improving the function and mechanism of the *Lin28* family in the formation of PGCs.

**Abstract:**

*Lin28A* and *Lin28B* are two homologues of the same family of RNA binding proteins (RBPs). The function and molecular mechanism of *Lin28A* in the formation of primordial germ cells (PGCs) are very clear, but the related research on *Lin28B* is rarely reported. Here, we found that the overexpression of *Lin28B* can promote the formation of PGC in vivo. Furthermore, the overexpression of *Lin28B* also resulted in the inhibition of totipotency gene expression and upregulated the PGCs marker genes, and a significant increase in the number of PGCs in genital ridge, as detected by Periodic Acid-Schiff(PAS) staining. However, the inhibited *Lin28B* expression showed completely opposite results, which were confirmed on the PGC induction model in vitro. Mechanistically, we found that the overexpression of *Lin28B* can inhibit the maturation of *let-7a-3p*, and the results of high-throughput sequencing indicated that *let-7a-3p* was a negative regulator of the formation process of PGCs. Therefore, we conclude that our results determine that *Lin28B* participates in the formation of PGCs through *let-7a-3p*, which set a theoretical foundation for improving the function and mechanism of *Lin28* family in the formation of PGCs.

## 1. Introduction

The function of *Lin28* is primarily concentrated on embryo development [1,2] and cell reprogramming [3,4], which could code two RNA-binding proteins, *Lin28A* and *Lin28B* [5]. As early as 2009, it was shown that *Lin28A* played an important role in the cytogenesis of primary germ cells (PGCs) [6]. Particularly, *Lin28A* inhibits the maturation of *let-7*, thereby activating the expression of the *Blimp1* gene (key gene for the formation of PGCs) [7,8]. However, the function of *Lin28B* in the formation of PGCs remains unknown. Recently, several research groups have found that the SNP locus in *Lin28B* is closely related to the age of female menarche [9,10,11], indicating the close correlation of *Lin28B* in animal reproduction. 

Subsequently, increasing studies observed the function of *Lin28A* and *Lin28B* in the PGCs development of mammals. The proliferation of PGCs in mice with *Lin28A* knockout was impaired, leading to decreased number of germ cells during embryo development [12]. *Lin28A* knockdown during the differentiation of ESCs into PGCs could significantly reduce the expression of PGCs-formation related genes, such as *Blimp1*, *Prdm14*, and *Stella* [13]. Similar to *Lin28A*, the knockdown of *Lin28B* could impair the formation of PGCs that are derived from Embryoid Bodies (EBs). The knockdown of *Lin28B* in ESCs could inhibit the differentiation of ESCs into PGCs [8]. Contrastingly, the expression pattern of *Lin28A* and *Lin28B* in the reproductive system is not always consistent. When there are only PGCs in genital ridge, the expression level of *Lin28A* is the highest. With the progress of pregnancy, the expression level of *Lin28* decreased significantly, but that of *Lin28B* did not change markedly [14,15], which makes the function of *Lin28B* in PGCs uncertain.

Although *Lin28A* and *Lin28B* are homologies, they bind to target miRNA and play a major role in post-transcriptional control [16,17]. *Lin28A* avoids Dicer processing by inducing the 3′ end of pre-*let-7* into uraci, and, finally, degrades it [18]. Contrastingly, *Lin28B* is a posttranscriptional inhibitor of pre-*let-7*, it binds to the end of pre-*let-7* through its cold shock domain(CSD) and to the GGAG motif of pre-*let-7* through its zinc finger domains(ZFD) [19,20]. This raises a question regarding whether totally different mechanism will lead to totally different function. For this reason, the function and underlying molecular mechanisms of *Lin28B* in the formation of PGCs were comprehensively studied while using chicken PGCs as the study objects. The results of the study may lay foundations for analyzing the function and mechanism of *Lin28B* in the formation of chicken PGCs. 

## 2. Materials and Methods 

### 2.1. Ethics Statement

All of the procedures involving the care and use of animals conformed to the U.S. National Institute of Health guidelines (NIH Pub. No. 85-23, revised 1996) and they were approved by the Laboratory Animal Management and Experimental Animal Ethics Committee of Yangzhou University. 

### 2.2. Reagents 

BMP4 (cyt-361) was from PROSPEC (Beijing, China). Dulbecco’s modified eagle medium (DMEM, 41965062) and fetal bovine serum (FBS, 10100-147) were supplied by Gibco (Carlsbad, CA, USA). The transfection reagents FuGENE^®®^HD (E2311) and Dual-Luciferase^®®^ reporter assay system were from Promega (Madison, WI, USA). The PrimeSTAR^®®^ Max DNA polymerase, the reverse-transcription kit (RR036A), the quantification kit for qRT-PCR(RR820A), and the restriction endonucleases SnaB I, Kpn I, and Xho I were supplied by Takara (Takara, Dianlian, China). The CVH (DDX4) antibody (ab27591) and the CKIT antibody (ab5634) were from abcam (San Francisco, CA, USA). 

### 2.3. Cell Treatments and Grouping

We studied the role of *Lin28B* in the formation of PGCs in vitro while using the BMP4 induction model that was established previously. The isolation and cultivation of ESCs are based on previous study [21]. Well-grown ESCs were transferred to 24-well plates and then treated/grouped, as follows. The routinely induced BMP4 was used as a control. The ESCs that were transfected with oe*Lin28B* and si*Lin28B* vector then induced by BMP4 were the treatment group. During induction, the culture media were replaced every two days. Cell morphology was checked with a fluorescence inversion microscope system. The zero-day-old, two-day-old, four-day-old, and six-day-old cells were collected for later analysis. The in vivo experiment was performed, as follows. The in vivo experiment was performed, as follows: the vectors of oe*Lin28B* and si*Lin28B* were mixed with PEI (M:V = 1:1), respectively, and then the mixture was injected into the blood vessels of 2.5 day-old chicken embryos with 1µg. The oe*Lin28B* group and si*Lin28B* group were the treatment group, and chicken embryos were checked every two days. The genital ridges of 4.5-day-old chicken embryos were collected for later analysis. 

### 2.4. Construction of Lin28B Overexpression Vector

The forward and reverse primers were designed while using the Primer 5.0 software according to the sequence of chicken *Lin28B* in GenBank (accession no.: NM_001034818.1). The forward primer was (F):5′-cgggatccATGGCCGAAGCAGGGGC-3′ and the reverse primer was (R):5′-cggaattcCGCACA TGACACA-3′. The enzyme digestion sites were BamH I and EcoR I. The total RNA of 4.5-day-old reproductive ridge was extracted and then reverse-transcripted to cDNA. With the cDNA as a template, clonal fragments of the target gene *Lin28B* were obtained through the use of PCR. The conditions for PCR were three min. at 98 °C, 25 s at 98 °C, 30 s at 64 °C, and 1 min. at 72 °C. The PCR cycles numbered 35 with an extension time for 7 min. at 72 °C. The linear plasmid pcDNA3.1 after dual-enzyme digestion and amplified target gene fragments of *Lin28B* were ligated in order to construct the *Lin28B* overexpression vector, oe*Lin28B*.

### 2.5. Construction of the Lin28B Interference Vector

We designed three targets according to the sequence of *Lin28B* and synthesized a single-strand RNA (Supplementary Table S1). After being annealed, the RNA was ligated to the lentivirus interference vector skeleton of piLenti-siRNA-GFP to construct four overexpression interference vectors of *Lin28B*, called si*Lin28B*-1, si*Lin28B*-2, si*Lin28B*-3, and si*Lin28B*-4. These four vectors were then transfected to confluent DF-1 cells, according to the mixture of plasmids and FuGENE HD at a ratio of 1:3 (M/V). Forty-eight hours later, the transfected DF-1 cells were selected with 10 ng/µL of puromycin for 24 h, after which the expression of green fluorescence proteins was observed with a fluorescence microscope. The DF-1 cells were collected from every group, and their total RNA was extracted with the Trizol method. The cDNA was synthesized through the use of a reverse-transcription kit, and the relative expression level of *Lin28B* was assayed with qRT-PCR. The amount of expression was calculated according to the 2^−ΔΔCt^ method, and β-actin was the internal reference gene.

### 2.6. qRT-PCR

Zero-day-old, two-day-old, four-day-old, and six-day-old cells during in vitro induction as well as the zero-day and 4.5-day-old in vivo induction cells from each group were collected. The total RNA was extracted with the Trizol method and it was transcribed to cDNA. The expression of the *NANOG* and the marker genes of PGCs genesis, such as DEAD-box helicase 4(*DDX4*, aslo called *Cvh*), Chicken tyrosine kinase receptor(*C-kit*), and PR domain 1(*Prdm1*, aslo called *Blimp1*), was determined with *β-actin* as the internal reference gene. qRT-PCR assay was based on the reverse-transcription kit from Tiangen (production no.: FP215). The 20 µl of reaction system includes 2 µl of cDNA (50 ng), 10 µl of 2 ×SuperReal Color PreMix, 0.6 µl of the forward and reverse primers (10 μM), and ddH2O. The reaction procedure of PCR is 15 min. at 95 °C, 10 s at 95 °C, and 32 s at 62 °C for 40 cycles. The expression was analyzed with the Ct values, and the primes are supplied in Supplementary Table S2. 

### 2.7. PAS Staining

4.5-day-old chicken embryos from each group were collected and fixed for 24 h. The fixed chicken embryos were then treated with 79% alcohol overnight, 70% alcohol for 1 h, 80% alcohol for 1 h, 90% alcohol for 1 h, 100% alcohol for 1 h, xylene for 10 min., and then xylene for an additional period of 10 min. The resulting chicken embryos were then immersed into 65 °C wax for 1 h and they were cooled down. Subsequently, the chicken embryos were sectioned at 8 μm, dewaxed, hydrated, and then subjected to glycogen staining with the PAS kit (Solarbio, Beijing, Chinas; product no.: G1281). 

### 2.8. Screening and Testing of Lin28B-Binding micRNA

The online software (http://mirdb.org/cgi-bin/search.cgi) is used in order to predict chicken micRNA *Let7*s. Well-grown DF-1 cells were transfected with oe*Lin28B* and si*Lin28B*, respectively. 48 h later, the transfected cells were collected and the total RNAs were extracted according to the miRNA isolation kits (thermofisher, Shanghai, Chinas) and then transcribed into cDNA, according to the manufacturer’s recommendations. The relative expression level of micRNA-*let7*s was assayed while using *U6* as an internal reference. Supplementary Table S3 lists the primers for qRT-PCR of micRNA-let7s. The condition for qRT-PCR was, as follows, according to the reagent kit. The reaction system includes 50 ng cDNA, 10 µL of 2 × miRucte Plus miRNA PreMix, 0.4 µL of forward primer, 0.4 µL of reverse primer (10 μM), and ddH2O. The entire reaction volume is 20 µl. The procedure for PCR is 15 min. at 95 °C, 20 s at 94 °C, 30 s at 63 °C, and 34 s at 72 °C for five cycles, followed by 20 s at 94 °C and an extension at 60 °C for 34 s. The total number of cycles was 40. The expression was analyzed with the Ct values. 

### 2.9. Immunocytochemical Detection of Reproductive Marker Protein

All collected groups of ESCs were cultured for six days, washed twice with PBS, fixed with 4% paraformaldehyde for 30 min., washed three times with PBS, treated with 0.1% Triton for 15 min., washed three times with PBS, and then added 10% FBS-PBS. After blocking for 2 h, added primary antibody CVH, CKIT, incubated for 2 h at 37 °C and overnight at 4 °C; washed primary antibody with PBS, added secondary antibody, incubated for 2 h at 37 °C in the dark; after that, washed secondary antibody with PBS, incubate for 15 min., stained with 5 ng/μL DAPI, blocked with glycerol (50% glycerol, 50% PBS).

### 2.10. Data Analysis

qRT-PCR were replicated three times. The data were analyzed by ANOVA with SPSS 19.0 software package (SPSS, Chicago, IL, USA). The means were compared by the least significant difference (LSD) test. Each replication was an experiment unit. *p* < 0.05 was considered to be significant, and *p* < 0.01 was highly significant. The charts were prepared in GraphPad Prism 6 (GraphPad Software Inc., San Diego, CA, USA).

## 3. The Results

### 3.1. Subsection Construction of Overexpression and Interference Vector of Lin28B

**Figure 1:**
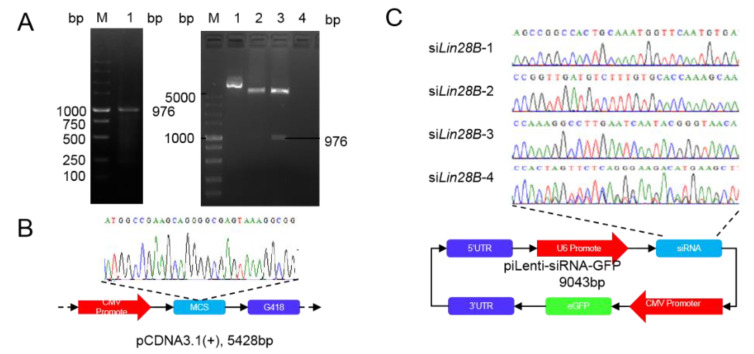
Construction of overexpression and interference vector of *Lin28B*. (**A**) Left: cloning results of *Lin28B*,1: *Lin28B* amplification bands, M: DL5000 marker; Right: double digestion results of *Lin28B* overexpression vector, M: DL 5000 marker; 1: no enzyme digestion; 2: single enzyme digestion; 3: double enzyme; 4: ddH_2_O. (**B**,**C**) Construction schematic diagram of *Lin28B* overexpression and interference vector.

**Figure 2:**
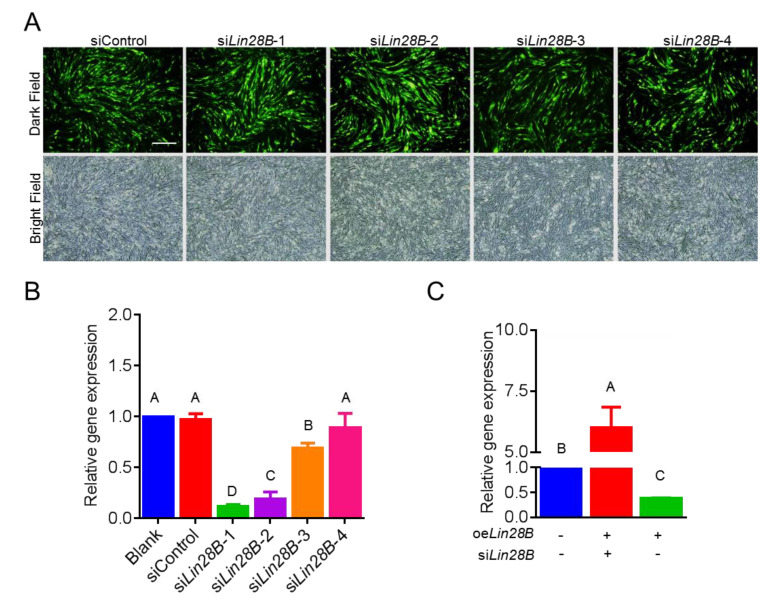
Activity detection of overexpression and interference vector of *Lin28B.* (**A**) *Lin28B* interference vector transfected into DF-1 cells, non-transfected cells were blank control, Scale bar: 50μm. (**B**) qRT-PCR was used to detect the relative *Lin28B* gene expression following *siLin28B*1-4 vector transfection. (**C**) Activity detection of *Lin28B* overexpression and interference vector by the rescue experiment. Different uppercase letters represent highly significant, and the same letters represent no significant.

The CDS region (979 bp long) of *Lin28B*(NM_001034818.1) was successfully amplified while using PCR. The results of agar gel electrophoresis showed there were specific bands at approximately 1000 bp (Figure 1A, Left). The amplified fragment was ligated to pcDNA3.1 in order to construct an overexpression vector of *Lin28B*, and the results of dual-enzyme digestion showed that there were two bands at 979 bp and 5400 bp (Figure 1A, Right). The sequencing results showed that the amplified product of 979 bp had a 99% similarity to the coding area of *Lin28B*, thus indicating that the *Lin28B* vector was successfully constructed, and the vector was named oe*Lin28B* (Figure 1B). The interference vector of *Lin28B* was constructed while using piLenti-siRNA-GFP as the skeleton (Figure 1C). The sequencing results confirmed that the expression interference vector of *Lin28B* was successfully constructed (Figure 1C), and the vectors were named si*Lin28B*-1, si*Lin28B*-2, si*Lin28B*-3, and si*Lin28B*-4. The expression interference vectors were transfected into DF1 cells (Figure 2A). The results showed that the transfection efficiency of these expression interference vectors in DF1 was greater than 70%. The results of qRT-PCR showed that si*Lin28B*-1, si*Lin28B*-2, and si*Lin28B*-3 could significantly reduce the expression of *Lin28B* (*p* < 0.01), and the expression levels were reduced by 88%, 62%, and 31%, respectively (Figure 2B). Moreover, si*Lin28B*-1 had the highest efficiency in interfering with the expression of *Lin28B*, whereby it was named si*Lin28B*. Meanwhile, the rescue experiment further showed that oe*Lin28B* overexpression in DF-1 markedly rescued the expression of *Lin28B* following si*Lin28B* interference (Figure 2C). The results suggest that oe*Lin28B* and si*Lin28B* could both overexpress and interfere with the activity of *Lin28B*.

### 3.2. Lin28B Overexpression/Interference Could Promote/Inhibit PGCs Formation In Vivo

**Figure 3:**
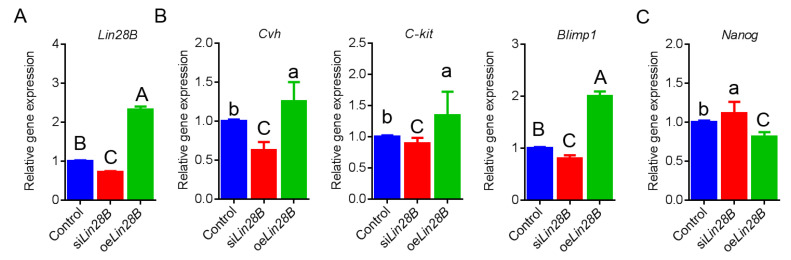
Lin28B overexpression/interference could promote/inhibit the formation of PGCs in vivo. (**A**–**C**) qRT-PCR was used to detect the expression of Lin28B, primary germ cells (PGCs) marker genes (Cvh, Ckit, and Blimp1), and totipotency marker gene (*NANOG*) in vivo. Different uppercase letters represent highly significant, different lowercase letters represent significant, and the same letters represent not significant.

**Figure 4:**
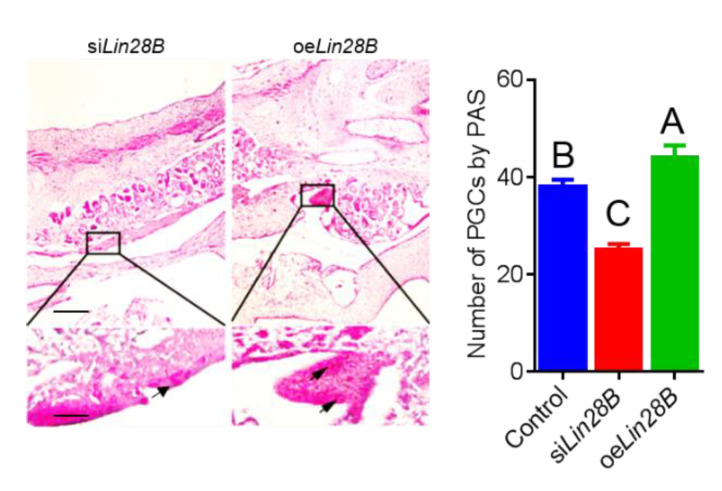
Quantity detection of PGCs by Periodic Acid-Schiff(PAS) staining; up-scale bar: 200 μm; down-scale bar: 40 μm. Left: the PGCs were marked by arrow, Right: statistical analysis of the number of PGCs in genital ridge after overexpression or interference of *Lin28B*. Different uppercase letters represent highly significant, and the same letters represent no significant.

In order to investigate the role of *Lin28B* in the formation of PGCs, oe*Lin28B* and si*Lin28B* were injected into chicken embryos incubated for 2.5 days (E2.5, HH14) through embryo blood vessels, and were then incubated for 4.5 days after injection. qRT-PCR detected the expression of PGCs related genes in the genital ridges of chicken embryos that were incubated for 4.5 days. The results showed that, when oe*Lin28B* was injected into the genital ridge of the chicken embryo, *Lin28B* gene expression was significantly up-regulated. Contrastingly, when si*Lin28B* was injected into the genital ridge of the chicken embryo, *Lin28B* gene expression was significantly down-regulated. This suggests that oe*Lin28B* and si*Lin28B* could both be expressed in chicken embryo (Figure 3A). Further gene expression analysis showed that, when *Lin28B* was overexpressed, the PGC makers *Cvh*, *C-kit*, and *Blimp1* were significantly up-regulated, while the totipotency marker gene *NANOG* was significantly down-regulated (Figure 3B,C). Contrastingly, a contrary result was observed when *Lin28B* was inhibited. In order to further confirm the regulating role of *Lin28B* in the formation of PGCs, we collected 4.5-day-old chicken embryos with different *Lin28B* treatments, prepared paraffin slices, and quantified the change in numbers of PGCs in the genital ridges through PAS staining. The results showed that the number of PGCs in the genital ridge of the si*Lin28B* group was significantly reduced when compared to the control group (25 ± 1.25 vs. 38 ± 1.53, *p* < 0.01). Contrastingly, the number of PGCs in the genital ridges of the oe*Lin28B* group was significantly increased (44 ± 2.56, *p* < 0.01) (Figure 4). In conclusion, during the formation of PGCs in the chicken embryos, PGC proliferation within the germinal ridge during chicken embryo development is significantly altered when overexpressing or inhibiting *Lin28B* expression.

### 3.3. Lin28B Could Promote Formation of EB in Bmp4-Induced Model In Vitro

**Figure 5:**
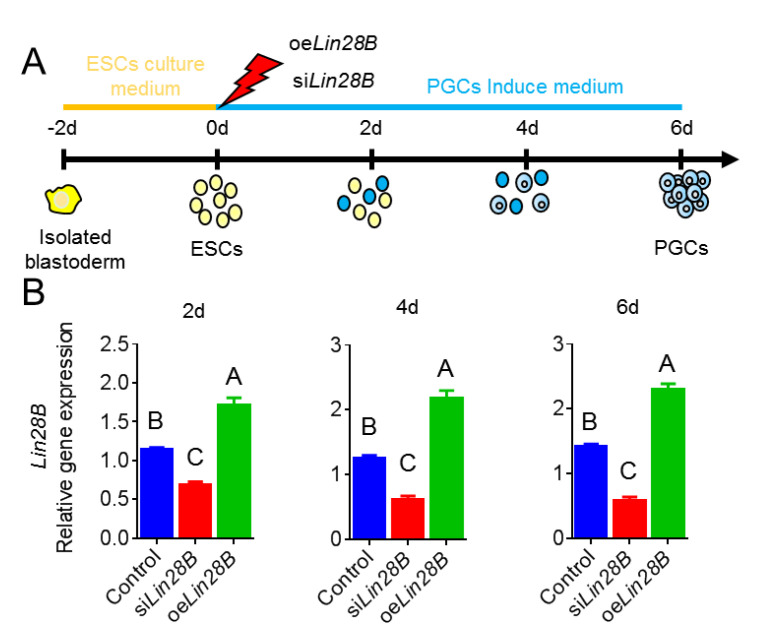
Lin28B could promote the formation of embryoid bodies(EBs) in BMP4-induced model in vitro. (**A**) Schematic diagram of illustrating the *Lin28*B function via PGCs induction model in vitro. (**B**) Expression detection of *Lin28B* during the induction of PGCs. Different uppercase letters represent highly significant and the same letters represent no significant.

**Figure 6:**
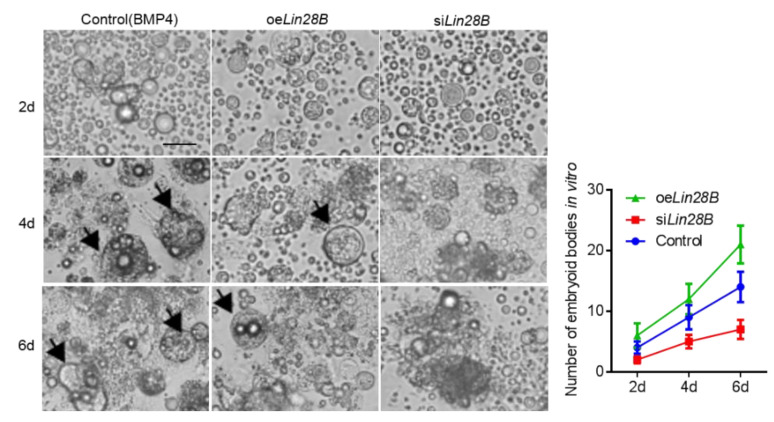
Observation (**Left**) and statistics (**Right**) of the number of EBs during induction after different treatments of *Lin28B*. Scale bar: 60 μm.

It is well known that ESCs could be induced into PGCs in vitro [22]. In order to further confirm the function of *Lin28B* in the formation of PGCs, we transfected oe*Lin28B* and si*Lin28B* into ESCs, respectively, and then induced by BMP4 (Figure 5A). The results of qRT-PCR showed that oe*Lin28B* and si*Lin28B* could overexpress/inhibit the expression of *Lin28B* during PGCs formation (Figure 5B). The results of morphological statistics showed that cells expanded at day 2 after BMP4 induction (control group), a small number of EBs appeared at day 4, and more EBs appeared at day 6. However, EB was not observed from day 2 to day 6 after *Lin28B* interference during BMP4 induction. Contrastingly, small EBs were observed at day 2, more EBs were observed and began to break at day 4, and, at day 6, the edge of EBs began to break and small amounts of cells were released from the EBs (Figure 6). This suggests that *Lin28* can regulate the formation of EBs.

### 3.4. Lin28B Could Positively Modulate Formation of PGCs in Bmp4-Induced Model In Vitro

**Figure 7:**
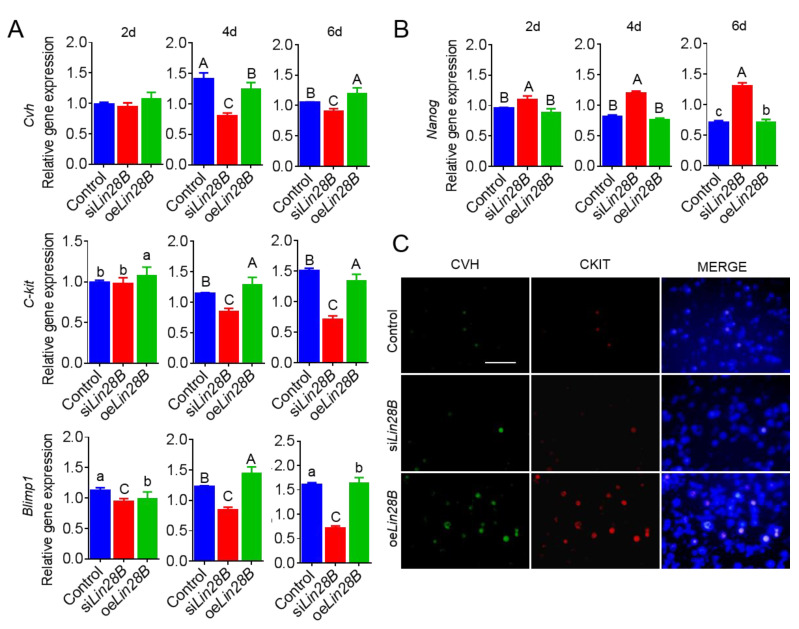
Lin28B could positively modulate the formation of PGCs in BMP4-induced model in vitro. (**A**,**B**) qRT-PCR was used to detect the expression of *NANOG*, *Cvh*, *Ckit*, and Blimp1 during the induction of PGCs *in vitro*. Different uppercase letters represent highly significant, different lowercase letters represent significant, and the same letters represent no significant. (**C**) Indirect immunofluorescence detection of the efficiency of the formation of PGCs at day 6 in the induced model after treatment with different Lin28B *in vitro*. Scale bar: 60 μm.

In order to confirm the effects of *Lin28B* on BMP4 induction, cells in different induction period were collected to detect the expression of *NANOG*, and marker genes of PGCs, such as *Cvh*, *C-kit*, and *Blimp1* (Figure 7A,B). The results showed that *NANOG* expression did not differ significantly from the normal BMP4 induction (control) at day 2, but it was significantly up-regulated at day 4 and day 6 (*p* < 0.01) after *Lin28B* inhibition. The expression of marker genes of PGCs was markedly reduced at day 4 and day 6 (*p* < 0.01). Contrastingly, although *NANOG* was down-regulated after *Lin28B* overexpression, no significant difference was observed when compared to the control. The marker genes of PGCs were not significantly expressed at day 2 after *Lin28B* overexpression as compared to the control, but they were markedly up-regulated at day 4 and day 6 (*p* < 0.01). The six-day-old cells were subjected to an indirect immunofluorescence test (Figure 7C). The results showed that the proportion of CVH + CKIT+ was significantly increased after *Lin28B* overexpression as compared to the control. Contrastingly, the proportion of CVH + CKIT+ was reduced significantly after *Lin28B* inhibition. In conclusion, the inhibition of *Lin28B* could significantly inhibit the formation of PGCs, while *Lin28B* overexpression could significantly promote the formation of PGCs.

### 3.5. Lin28B Promotes Formation of PGCs Through Inhibition of gga-Let-7a-3p

**Figure 8:**
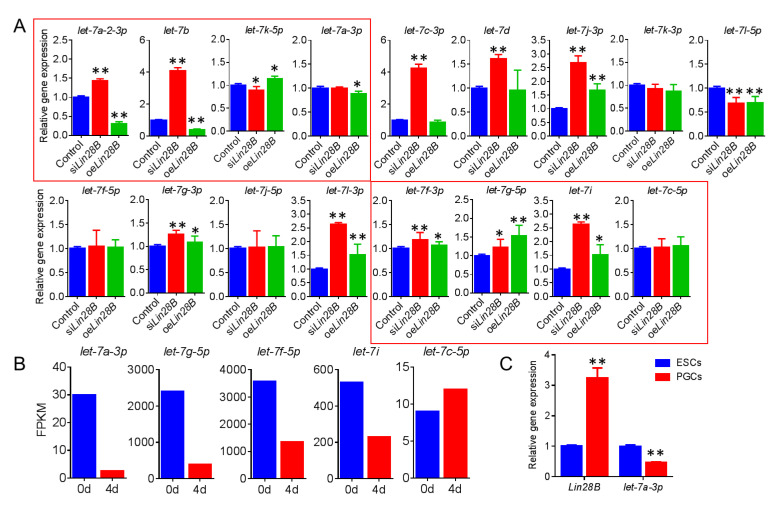
*Lin28B* promotes the formation of PGCs through the inhibition of *gga-let-7a-3p*. (**A**) qRT-PCR was used to detect the expression of *let-7* family after overexpression and inhibition of Lin28B, only *let-7a-2-3p*, *let-7a-3p*, let-7b, and *let-7k-5p* showed opposite trend to Lin28B. (**B**) miRNA sequencing to screen *let-7* (*let-7a-3p*, *let-7g-5p*, *let-7f-5p*, *let-7i*, and *let-7c-5p*) during the formation of PGCs in vitro. (**C**) qRT-PCR was used to detect the expression of *let-7a-3p* and *Lin28B* during the generation of PGCs in vivo. “*” means *p* < 0.05, and “**” means *p* < 0.01.

The study results showed that *Lin28B* functions by binding the *let7* family [8]. A total of 17 gga-let7s were predicted in chicken by online software (method for details) in order to investigate the key micRNA *let7s* targeted by *Lin28B* (Figure 8A). We transfected si*Lin28B* and oe*Lin28B* in DF-1 cells, and then detected the expression of these micRNAs with qRT-PCR. We found that only *let-7a-2-3p*, *let-7a-3p*, *let-7b*, and *let-7k-5p* of the 17 *gga-let7s* were significantly regulated by *Lin28B* (the expression trend is opposite to *Lin28B*) (Figure 8A). Additionally, we collected zero-day and four-day-old cells in the PGC induction model in vitro [22] and performed transcriptome sequencing (data not published), and found that there are five *gga-let7s*(*let-7a-3p*, *let-7g-5p*, *let-7f-5p*, *let-7i*, and *let-7c-5p*) in the process of the formation of PGCs *in vitro*, of which the expression of *let-7a-3p*, *let-7g-5p*, *let-7f-3p*, and *let-7i* showed a downward trend, which was consistent with the expression rule of gga-let7s during the formation of PGCs (Figure 8B). However, we found that after the overexpression of *Lin28B*, l*et-7g-5p*, *let-7f-3p*, and *let-7i* showed an upward trend, which means that, in addition to *let-7a-3p*, the maturation process of *let-7g-5p*, *let-7f-3p*, and *let-7i* is not regulated by *Lin28B*. Moreover, we examined the expression of *let-7a-3p* in ESCs and PGCs, and found that *let-7a-3p* was significantly down-regulated, which showed a completely opposite trend to the expression of *Lin28B* (Figure 8C). Combining these results, we therefore conclude that *Lin28B* regulates the formation of chicken PGCs through *let-7a-3p*.

## 4. Discussion

The research study deeply examined the function of *Lin28B* in the formation of PGCs, being screened and obtained the micRNA modulated by *Lin28B*, and preliminarily clarified the molecular mechanism by which *Lin28B* participates in the process of PGC genesis by inhibiting the maturation of *gga-let-7a-3p*. 

Despite that both *Lin28A* and *Lin28B* are from the same RBP family, more research has focused on *Lin28A*. As early as 2009 [6], it was shown that *Lin28A* could regulate the PGC genesis of mammals. The underlying molecular mechanism is that *Lin28A* could activate the expression of *Blimp1* to participate in the formation of PGCs by inhibiting the maturation of *let-7*’s precursor [18]. However, no relevant study concerning *Lin28B* has ever been reported. The function of *Lin28B* in PGC genesis was only putatively predicted by in vitro induction experiment or by addressing the association between SNP loci and menstrual cycle, because there are SNP loci in *Lin28B* [14,23]. However, no definite conclusion has been drawn. In the subject study, we systematically examined the function of *Lin28B* in the genesis of PGCs while using the chicken as a model animal. The results showed that, similar to *Lin28A*, *Lin28B* could also regulate PGCs genesis. Although the study object of the study is chicken, the results could serve as the foundation for understanding the specific function and mechanism of *Lin28B* gene family in PGCs genesis. Based on the results of this study, we can improve the regulatory role of *Lin28* family in the formation of chicken PGCs, laying a foundation for the application of chicken PGCs in the production of transgenic animals, resource protection, and other fields

With the in-depth study in Lin28, increasing numbers of scholars have started to examine the functioning mechanism of *Lin28* in totipotency, genesis of germ cells, and tumorigenesis [1,4,24]. The results showed that *Lin28A* and its collateral derivative *Lin28B* are the key participants for *Let-7* processing [25], which functions by modifying its processing or stability through binding to *Let-7* pre-miRNA or pri-miRNA. [26] Nie et al. [27] found the link between *Blimp1* and *Let7a* in Hodgkin’s lymphoma cells. They found that *Let-7* was highly expressed in Reed–Sternberg cells, whereas *Blimp1*, a primary regulator for the differentiation of B cells, was significantly inhibited. The binding of *Let-7a* miRNA to the target site in 3′UTR of *Blimp1* was further observed, which could inhibit *Blimp1* expression. This undoubtedly proven that *Lin28* indirectly regulates *Blimp1*. It was not until 2009 when West et al. [6] confirmed that link between *Lin28* and *PGCs*. West et al. knocked out *Lin28A* or *Lin28B* in the ESCs of Stella-GFP, which led to a small number of positive TNAP communities (an early marker of PGCs). They assumed that *Lin28A* and *Lin28B* could inhibit the expression of *Blimp1* by inhibiting the expression of *Let-7*. In fact, the deletion of the *let-7* locus in *Blimp1* 3′UTR can rescue the phenotypic loss of PGCs due to *Lin28* knockdown. In addition, an overexpression of *Lin28* could induce the expression of more marker genes of TNAP-positive PGCs. Such findings confirmed the importance of *Lin28* in PGCs genesis; our results filled the gap in the study of Lin28B in the formation of PGCs in chickens. Early studies of *Lin28* and *Let-7* focused on mammalian PGCs formation, with few reports on chickens. Therefore, it is the first time we demonstrated the key role of *Let-7* miRNA biosynthesis in PGCs. We expanded this finding from mammalian to the poultry area, studied and analyzed the function of *Lin28B* in PGC genesis, and targeted it to *gga-let-7a-3p*. Our study may provide a constructive basis for future in-depth research. 

## 5. Conclusions

In this study, we confirmed that *Lin28B* promoted the formation of chicken PGCs through in vivo and in vitro experiments. The let-miRNA family that was opposite the expression of *Lin28B* was identified by combining the results of high-throughput sequencing, and it confirmed that *Lin28B* promoted the formation of chicken PGCs by inhibiting the maturation of gga-*let-7a-3p*.

## Data Availability

The data presented in this study are available on request from the corresponding author.

## Supplementary Materials

The following are available online at https://www.mdpi.com/2076-2615/11/1/43/s1, Table S1, Target sites sequence of Lin28B gene; Table S2 qRT-PCR primers sequence of related genes; Table S3, qRT-PCR primers sequence of related genes.
