# Supplementary material for: Study on the Function and Mechanism of Lin28B in the Formation of Chicken Primordial Germ Cells
Source: Animals (Basel). 2020 Dec 28;11(1):43. doi: 10.3390/ani11010043 (PMC7823903; doi:10.3390/ani11010043)
Supplement: Supplementary file 1 [file animals-11-00043-s001.zip › S Table2.docx]

S Table2 qRT-PCR primers sequence of related genes

| Gene | Primer Sequence（5'-3'） | GenBank ID |
| --- | --- | --- |
| *Nanog* | F:CGTCCTACGGCTCTGTTA | NM_001146142.1 |
|  | R:CCTTCCTTGTCCCACTCT |  |
| *Cvh* | F:AGGAGGACTGGGACACG | AB004836.1 |
|  | R:GCCTCTTGATGCTACCG |  |
| *C-kit* | F:GCGAACTTCACCTTACCCGATTA | NM_204361.1 |
|  | R:TGTCATTGCCGAGCATATCCA |  |
| *Blimp1* | F:ATGAAGGCTGCTACACGG | XM_004940353.2 |
|  | R:GCAGTTTGATGCGTATTTG |  |
| *β-actin* | F:CAGCCATCTTTCTTGGGTAT | L08165.1 |
|  | R:CTGTGATCTCCTTCTGCATCC |  |
