# Supplementary material for: Study on the Function and Mechanism of Lin28B in the Formation of Chicken Primordial Germ Cells
Source: Animals (Basel). 2020 Dec 28;11(1):43. doi: 10.3390/ani11010043 (PMC7823903; doi:10.3390/ani11010043)
Supplement: Supplementary file 1 [file animals-11-00043-s001.zip › S Table3.docx]

S Table3 qRT-PCR primers sequence of related genes

| miRNA | Primer sequence (5'-3 ') |
| --- | --- |
| gga-let-7a-2-3p | GCCGAGCTGTACAACCTCCTAGCTTTCC |
| gga-let-7a-3p | GCCGAGCTATACAATCTACTGTCTTTCC |
| gga-let-7b | GCCGAGTGAGGTAGTAGGTTGTGTGGTT |
| gga-let-7c-3p | GCCGAGCTGTACAACCTTCTAGCTTTCC |
| gga-let-7c-5p | GCCGAGTGAGGTAGTAGGTTGTATGGTT |
| [gga-let-7d](http://www.mirbase.org/cgi-bin/mirna_entry.pl?acc=MI0001232) | GCCGAGAGAGGTAGTGGGTTGCATAGT |
| gga-let-7f-3p | GCCGAGCTATACAATCTATTGCCTTCCC |
| gga-let-7f-5p | GCCGAGTGAGGTAGTAGATTGTATAGTT |
| gga-let-7g-3p | CTGTACAGGCCACTGCCTTGCC |
| gga-let-7g-5p | GCCGAGTGAGGTAGTAGTTTGTACAGT |
| gga-let-7i | GCCGAGTGAGGTAGTAGTTTGTGCTGT |
| gga-let-7j-3p | GCCGAGCTATACAGTCTATTGCCTTCCT |
| gga-let-7j-5p | GCCGAGTGAGGTAGTAGGTTGTATAGTT |
| gga-let-7k-3p | GCCGAGCTATACAATCTACTGTCTTTCC |
| gga-let-7k-5p | GCCGAGTGAGGTAGTAGATTGAATAGTT |
| gga-let-7l-3p | CGATGCAGCCGACTACTTTCC |
| gga-let-7l-5p | GCCGAGTGAGGTAGTCGGTTGTATTGTT |
| U6 | F:TAAGCCTGGACTGAGTAAGAGCG |
|  | R:CCATATTAGAAGCCCCTTTTTGT |
