# Supplementary material for: Study on the Function and Mechanism of Lin28B in the Formation of Chicken Primordial Germ Cells
Source: Animals (Basel). 2020 Dec 28;11(1):43. doi: 10.3390/ani11010043 (PMC7823903; doi:10.3390/ani11010043)
Supplement: Supplementary file 1 [file animals-11-00043-s001.zip › S Table1.docx]

S Table1 Target sites sequence of *Lin28B* gene

| Name | Target sequence 5'-3' |
| --- | --- |
| siLin8B-1 | AGCCGGCCACTGCAAATGGTTCAATGTGA |
| siLin8B-2 | CCGGTTGATGTCTTTGTGCACCAAAGCAA |
| siLin8B-3 | CCAAAGGCCTTGAATCAATACGGGTAACA |
| siLin8B-4 | CCACTAGTTCTCAGGGAAGACATGAAGCT |
| siNC | GGGTGAACTCACGTCAGAAC |
